# Adaptive Contact Networks Change Effective Disease Infectiousness and Dynamics

**DOI:** 10.1371/journal.pcbi.1000895

**Published:** 2010-08-19

**Authors:** Sven Van Segbroeck, Francisco C. Santos, Jorge M. Pacheco

**Affiliations:** 1 COMO, Vrije Universiteit Brussel, Brussels, Belgium; 2 MLG, Département d'Informatique, Université Libre de Bruxelles, Brussels, Belgium; 3 CENTRIA, Departamento de Informática, Faculdade de Ciências e Tecnologia, Universidade Nova de Lisboa, Caparica, Portugal; 4 Departamento de Matemática e Aplicações, Universidade do Minho, Braga, Portugal; 5 ATP-Group, CMAF, Complexo Interdisciplinar, Lisboa Codex, Portugal; 6 GADGET, Lisboa, Portugal; Royal Holloway University of London, United Kingdom

## Abstract

Human societies are organized in complex webs that are constantly reshaped by a social dynamic which is influenced by the information individuals have about others. Similarly, epidemic spreading may be affected by local information that makes individuals aware of the health status of their social contacts, allowing them to avoid contact with those infected and to remain in touch with the healthy. Here we study disease dynamics in finite populations in which infection occurs along the links of a dynamical contact network whose reshaping may be biased based on each individual's health status. We adopt some of the most widely used epidemiological models, investigating the impact of the reshaping of the contact network on the disease dynamics. We derive analytical results in the limit where network reshaping occurs much faster than disease spreading and demonstrate numerically that this limit extends to a much wider range of time scales than one might anticipate. Specifically, we show that from a population-level description, disease propagation in a quickly adapting network can be formulated equivalently as disease spreading on a well-mixed population but with a rescaled infectiousness. We find that for all models studied here – **SI**, **SIS** and **SIR** – the effective infectiousness of a disease depends on the population size, the number of infected in the population, and the capacity of healthy individuals to sever contacts with the infected. Importantly, we indicate how the use of available information hinders disease progression, either by reducing the average time required to eradicate a disease (in case recovery is possible), or by increasing the average time needed for a disease to spread to the entire population (in case recovery or immunity is impossible).

## Author Summary

During the past decade, we learned that the structure of contact networks plays a crucial role in the spread of diseases. Most theoretical studies addressing this issue assume that contact networks are static entities, whereas the actual disease paths continuously reshape based on local social dynamics. This work aims to achieve a better understanding of disease spreading in populations characterized by a dynamically structured contact network where contacts appear and disappear over time. The network dynamics are entangled with the disease dynamics, as individuals may have access to local information that makes them aware of both the existence of the disease and the health status of their contacts, allowing them to minimize exposure to infection. Here we show the equivalence between disease propagation in an adaptive contact network and that in a well-mixed population with a rescaled transmission probability, which depends also on the fraction of infected in the population. Thus, one can emulate the effect of an adaptive contact network with a simple correction of the transmission probability. This result is obtained in the limit where network adaptation proceeds much faster than disease spreading, but we demonstrate that it also holds for a much wider range of scenarios.

## Introduction

During recent years it has become clear that disease spreading [1]–[3] transcends geography: the contact process is no longer restricted to the immediate geographical neighbors, but exhibits the stereotypical small-world phenomenon with inescapable impact [4]–[7]. The **SARS** and, at the time of writing, the influenza A outbreaks provide arresting examples of this effect in a biological context, although the spread of computer viruses constitutes perhaps the most obvious manifestation of such a property. Recent advances in the science of networks [2] also brought the impact of disease interactions beyond geography to the spotlight, providing compelling evidence of the role that the networks of contacts between individuals or computers play in the dynamics of infectious diseases [3], [5]. In the majority of cases in which complex networks of disease spreading have been considered [7], they were taken to be static entities. However, social networks are intrinsically dynamical entities. In fact, modern societies have developed rapid means of information dissemination, both at local and at centralized levels, which one naturally expects to alter individuals' response to vaccination policies, their behavior with respect to other individuals and their perception of likelihood and risk of infection [8]. In some cases one may even witness the adoption of centralized measures, such as travel restrictions [9], [10] or the imposition of quarantine spanning parts of the population [11], which may induce abrupt dynamical features onto the structure of the contact network. Furthermore, individual knowledge (based on local information) about the health status of acquaintances, partners, relatives, etc., combined with individual preventive strategies [12]–[20] such as condoms, vaccination, the use of face masks or prophylactic drugs, avoidance of visiting specific web-pages, staying away from public places, etc., may also lead to bias in the organization of disease paths along dynamical networks.

As a result, quite a few studies have recently investigated the impact of dynamical networks on disease progression, as well as the influence of the way information (disease awareness) flows in parallel with disease progression and the role of noise in disease dynamics [21]–[33]. At par with these models, our model studies disease dynamics in a finite and constant population of individuals. Contrary to current models though (see Discussion Section for more details), our dynamical contact structure allows for a variable number of overall links between individuals, which in turn depends on the overall incidence of disease in the population. This increased complexity of the model, however, will allow us to describe analytically disease dynamics in finite populations. Infection occurs along the links of a contact network whose structure may change based on each individual's health status and the availability of information regarding the health status of others. We assume the existence of some form of (unsupervised) local information about the health status of social contacts, depending on which individuals may be more or less effective in avoiding contact with those infected while remaining in touch with the healthy. We investigate some of the most widely used models of disease spreading, such as the popular Susceptible-Infected-Susceptible (**SIS**) model [1], [3], the Susceptible-Infected (**SI**) model [1] used to study, e.g., **AIDS** [1], [34], and the Susceptible-Infected-Recovered (**SIR**) model [1], [35], more appropriate to model, for instance, single season flu outbreaks [1] or computer virus spreading [5]. We provide analytical results which show that, on finite adaptive networks, the effective infectiousness of a disease depends on i) the size of the population, ii) the number of infected and ultimately iii) on the efficiency of local information spreading. These results, obtained under the assumption that network reshaping occurs much faster than disease spreading, are shown (numerically) to remain valid for a much wider range of time scales. When combined with the analytical results already known for static networks [3], which describe well the opposite limit in which the network dynamics is slow compared to disease dynamics, we demonstrate that one can describe analytically most of the time scale spectra. We further show how availability of information may either drastically reduce the time required to eradicate a disease (when recovery from the disease is possible), or drastically increase the time after which the entire population becomes infected (when the disease cannot be cured). Finally, we discuss the implications of assuming that individuals exhibit diverse response profiles to available information [36]–[38].

Let us start by introducing the well established **SIS** model [1], [3], whereby individuals can be in one of two epidemiological states: Infected (*I*) or Susceptible (*S*). Individuals occupy the nodes of a graph, whereas the links of the graph define who interacts with whom. As usual, we define a transmission probability λ and a recovery probability δ. We assume a population of finite and constant size *N*, but with variable number of links [39], [40]. Links are established and removed at rates which also depend on the health status of the individuals connected: Links of different types — *SS*, *SI* and *II* — will tend to last differently. The **SIR** model, on the other hand, requires the introduction of a new epidemiological state — Recovered (or Removed, *R*) — representing individuals who, once recovered from the infection, acquire immunity. In **SIR**, the same parameters λ and δ defined above remain finite, but now we have three new kinds of links — *SR*, *RI* and *RR*. Finally, the **SI** model can be viewed as the limit of the **SIS** model when 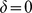, representing diseases from which recovery is impossible.

Suppose all individuals seek to establish links at the same rate *c*. For simplicity, we assume that new links are established and removed randomly, a feature which may not always apply in real cases, where the limited social horizon of individuals or the nature of their social ties may constrain part of their neighborhood structure (see Discussion). Let us further assume that links may be broken off at different rates, based on the nature of the links and the information available about the neighbors: Let us denote these rates by *b_pq_* for links of type *pq* (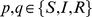). This allows us to write down a system of ordinary differential equations [39], [40] for the time evolution of the number of links of each type. These and all other technical details of the model are presented in the Methods section. In the steady state of the linking dynamics, the number of links of each type is given by 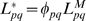, where 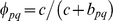 are the fractions of active *pq*-links, compared to the maximum possible number of links 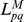, for a given number of *S*, *I* and *R*. In the absence of disease only *SS* links exist, and hence 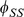 determines the average connectivity of the network under disease free conditions, characterizing the type of the population under study. In the presence of *I* individuals, to the extent that *S* individuals manage to avoid contact with *I*, they succeed in escaping infection. Hence, the intuition is clear: Reshaping of the contact network based on available information of the health status of individuals will contribute to inhibit disease progression. In the extreme limit of perfect information and individual capacity to immediately break up contacts with infected, we are isolating all infected, thus containing disease progression. Our goal here, however, is to understand how and in which way partial individual information affects the overall disease dynamics.

Often individuals prevent infection by avoiding unprotected contact with infected once they know the state of their contacts or are aware of the potential risks of such infection [12]–[20], [28], [32]: such is the case of many sexually transmitted diseases [12], [41]–[43], for example, and, more recently, the voluntary use of face masks and the associated campaigns adopted by local authorities in response to the **SARS** outbreak [11], [13]–[15]. In the present study, we assume that individuals are not centrally supervised or informed: Individual decision is based on available local information about the health state of one's contacts. To the extent that such information spreads quickly and contacts are not too frequent, one can study analytically the limit in which the network dynamics — resulting from adaptation to the flow of local information — is much faster than disease dynamics. In this case, one may separate the time scales between network adaptation and contact (disease) dynamics: The network has time to reach a steady state before the next contact takes place. Consequently, the probability of having an infected neighbor is modified by a neighborhood structure which will change in time depending on the impact of the disease in the population and the overall efficiency of local information flow. It will be shown that a quickly adapting community induces profound changes in the dynamics of disease spreading, irrespective of the underlying epidemic model. Furthermore, we will demonstrate numerically that the two limiting cases amenable to analytic treatment — static networks on the one hand, and quickly adapting networks on the other hand — remain valid for a wide range of intermediate time scales, strengthening the power of the analytical predictions derived here.

## Results

The amount of information available translates into differences mostly between the break-up rates of links that may involve a potential risk for further infection (*b_SI_*, *b_IR_*, *b_II_*), and those that do not (*b_SS_*, *b_SR_*, *b_RR_*). Therefore, we consider one particular rate *b_I_* for links involving infected individuals (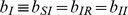), and another one, *b_H_*, for links connecting healthy individuals (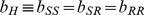). In general, one expects *b_I_* to be maximal when each individual has perfect information about the state of her neighbors and to be (minimal and) equal to *b_H_* when no information is available, turning the ratio between these two rates into a quantitative measure of the amount of information available or the efficiency of information use. Note that we reduce the model to two break-up rates in order to facilitate the discussion of the results. The general principles and conclusions remain valid when all break-up rates are incorporated explicitly. It is worth noticing that three out of these six rates are of particular importance for the overall disease dynamics: *b_SS_*, *b_SR_* and *b_SI_*. These three rates, combined with the rate *c* of creating new links, define the fraction of active *SS*, *SR* and *SI* links, and subsequent correlations between individuals [44], and therefore determine the probability for a susceptible to become infected (see Methods). This probability will increase when considering higher values of *c* (assuming *b_I_*>*b_H_*). In other words, when individuals create new links more often, therefore increasing the likelihood of establishing connections to infected individuals (when present), they need to be better informed about the health state of their contacts in order to escape infection. In the fast linking limit, the other three break-up rates (*b_II_*, *b_IR_* and *b_RR_*) influence disease progression since they contribute to altering the average degree of the network.

In the Methods, we show that disease spreading in a quickly adapting network can be studied *as if* it took place in a well-mixed population with same average degree 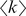 as the original network and transmission probability 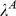. Since the lifetime of a link depends on its type, the average degree 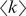 of the network depends on *the number of infected in the population*, and hence becomes frequency (and time) dependent. Similarly, fast linking dynamics leads to a rescaling of the transmission probability, 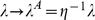, with 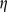 depending on the particular model of disease spreading. In the **SIR** model, 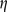 is given by
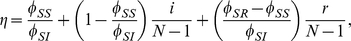
where *i* denotes the number of infected individuals in the population and *r* the number of recovered (immune). The rescaling parameter 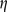 for the **SI** and **SIS** models is the same, but with 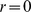. Note that 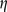 scales linearly with the frequency of infected in the population, decreasing the more individuals get infected (assuming 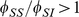), and depends implicitly (via the ratio 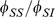) on the amount of information available.

We would like to stress the distinction between the description of the disease dynamics at the local level and that at the population level. Strictly speaking, a dynamical network does not change the disease dynamics at the local level, meaning that infected individuals pass the disease to their neighbors with probability intrinsic to the disease itself. At the population level, on the other hand, disease progression proceeds as if the infectiousness of the disease effectively changes, as a result of the network dynamics. Hence, analyzing an adaptive network scenario at a population level can be achieved via a correction on the transmission probability, keeping the mathematically more attractive well-mixed scenario. In this sense, from a well-mixed perspective, dynamical networks contribute to change the effective infectiousness of the disease, which becomes *frequency* and *information* dependent.

**Figure 1:**
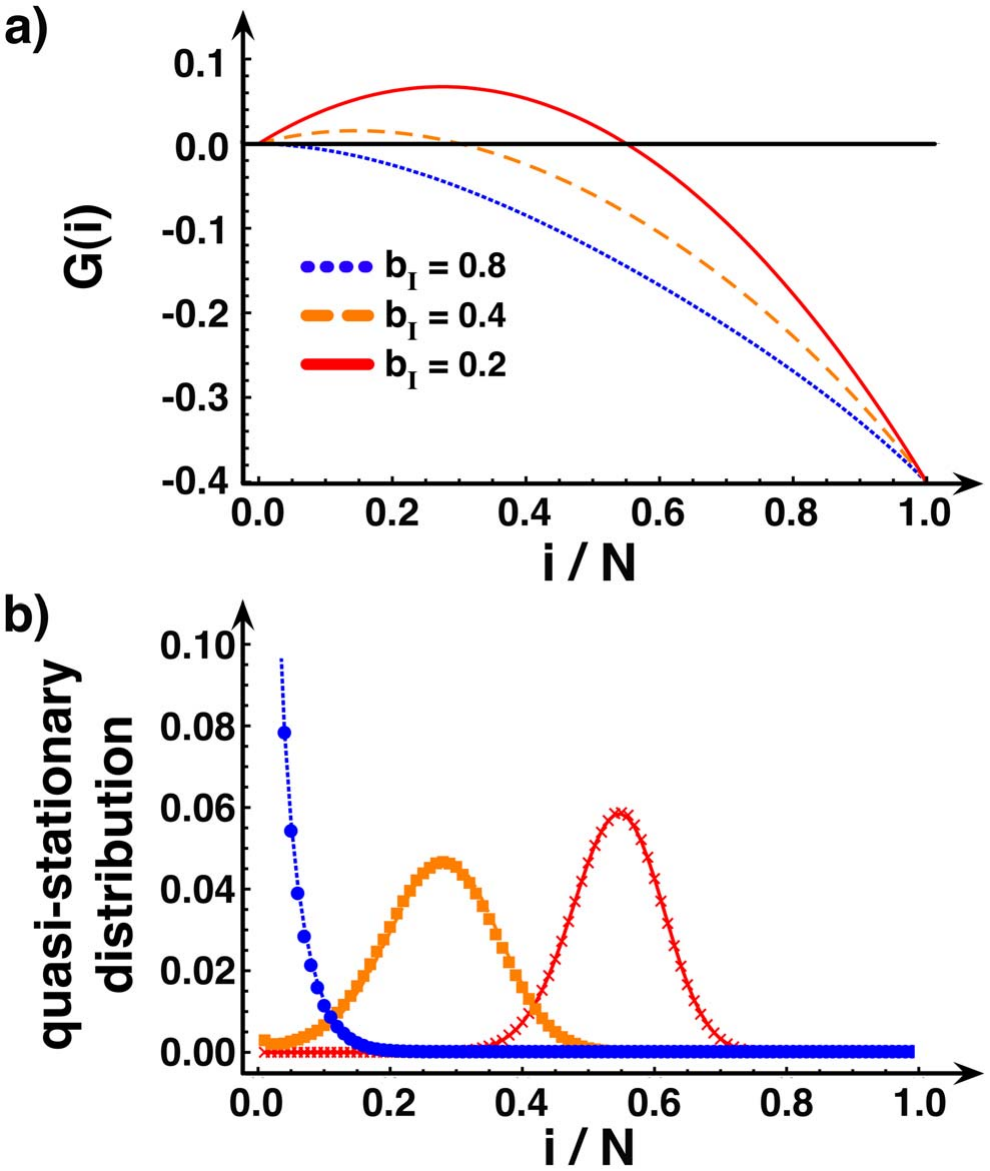
Disease spreading under fast linking dynamics in the SIS mode. The upper panel shows the gradient of infection *G* as a function of the fraction of infected for different values of the rate *b_1_* at which links with infected disappear (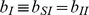): 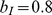 (dotted line), 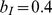 (dashed line) and 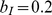 (solid line). The lower panel shows the corresponding quasi-stationary distributions, both analytically (lines) and via individual-based computer simulations (circles for 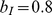, squares for 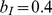 and crosses for 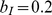). We use 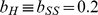, 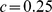, 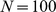, 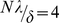 and 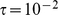.

One can define a gradient of infection *G*, which measures the tendency of the disease to either expand or shrink in a population with given configuration (defined by the number of individuals in each of the states *S*, *I* and *R*). For the **SIS** model, eradication of the disease is favored (*G(i)<0*), *irrespective of the fraction of infected*, whenever 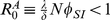 (see Eq. 35 in Text S1), indicating how the presence of information (*b_H_*<*b_I_*) changes the basic reproductive ratio. This is illustrated in the upper panel of Figure 1, which depicts *G* for different values of *b_I_* (assuming 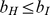) and a fixed transmission probability 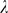. The corresponding quasi-stationary distributions, which characterize the relative time the population spends in each configuration (and defined in Methods), are shown in the lower panel and clearly reflect the sign of *G*. Whenever *G(i)* is positive (negative), the dynamics will act to increase (decrease), on average, the number of infected. Figure 1 indicates how the availability of local yet reliable information hinders disease progression: For 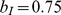 the interior root of *G(i)* disappears, making disease expansion unlikely in any configuration of the population.

**Figure 2:**
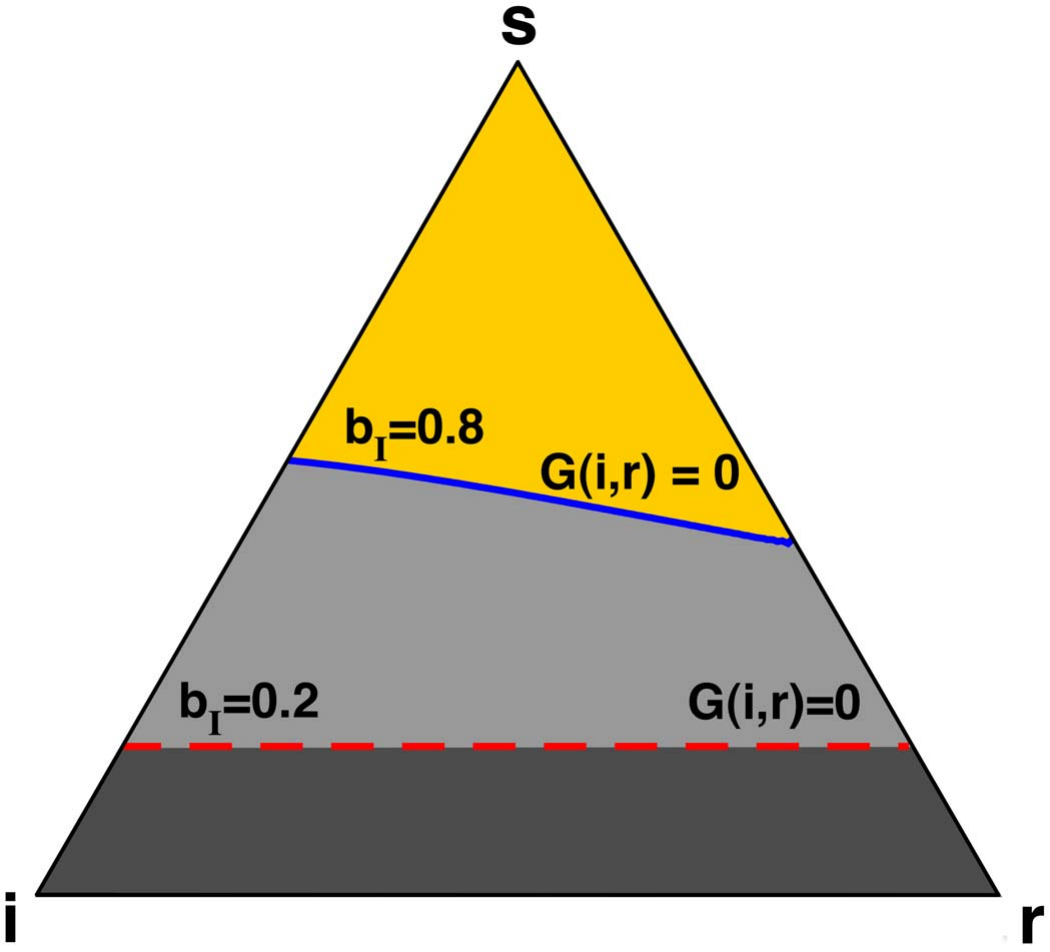
Gradient of infection in the SIR model in a network with information (solid blue line, 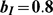, 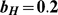), and without information (dashed red line, 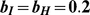). Each point in the triangle (the so-called *simplex*) satisfies that population size is conserved, e.g. *i+r+s* = *N*. Vertices of the simplex represent populations with only one class of individuals present. The colored lines in the interior of the simplex indicate configurations in which 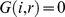. For each case, disease expansion is more likely than disease contraction in configurations above the line, and less likely otherwise, showing that availability of information greatly reduces the regions of state space in which disease may progress (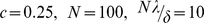).

The analysis of the gradient of infection of the **SIS** model has the natural advantage of showing the effect of adaptive networks in a one-dimensional simplex (the fraction of infected). Yet, an analogous result holds for the **SIR** model. The gradient of infection now also depends on the number of recovered (*r*) individuals in the population and, once again, allows us to identify when disease expansion will be favored or not. Figure 2 gives a complete picture of the gradient of infection, using the appropriate simplex structure in which all points satisfy the relation *i+r+s = N*. The dashed red line indicates where 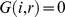 in case individuals do not have any information about the health status of their contacts, i.e., links that involve infected individuals disappear at the same rate as those that do not (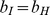). Disease expansion is more likely than disease contraction (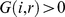) when the population is in a configuration above the line, and less likely otherwise. Similarly, the solid blue line indicates where 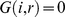 whenever individuals share information about their health status, and use it to avoid contact with infected. Once again, the availability of information modifies the disease dynamics, inhibiting disease progression for a broad range of configurations.

**Figure 3:**
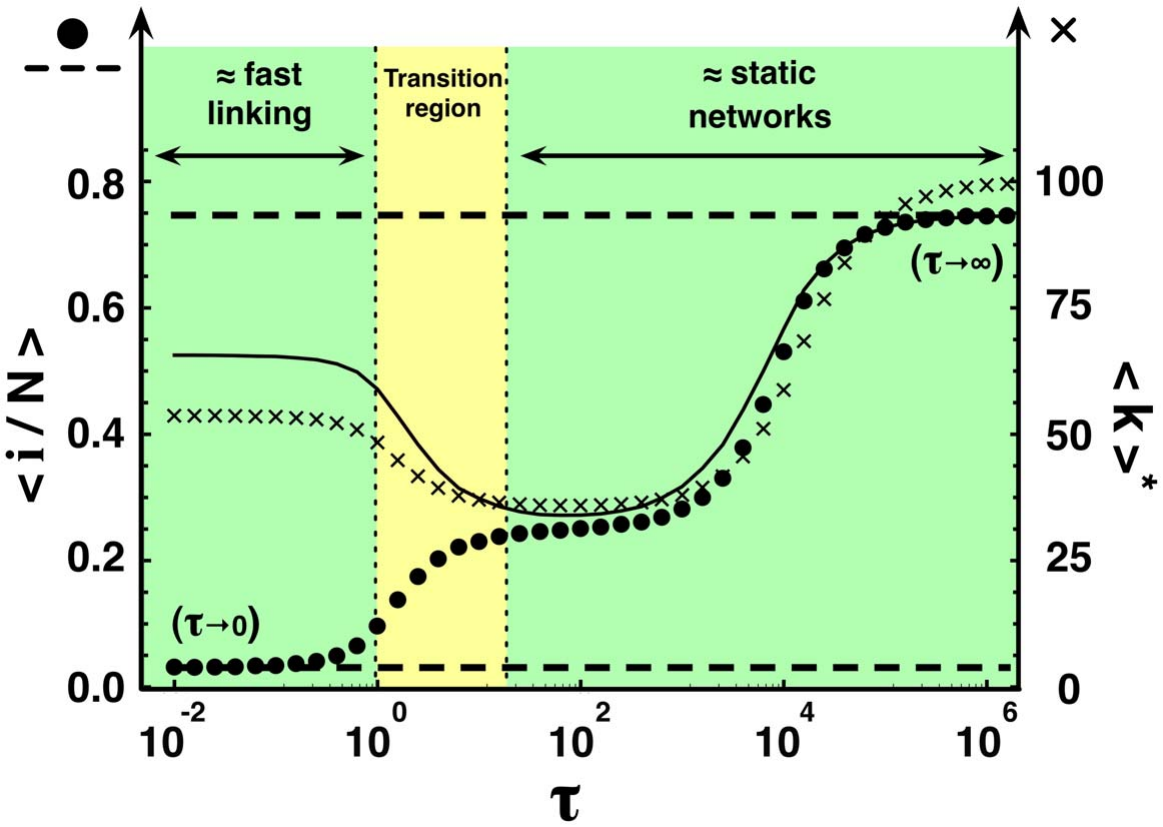
Disease spreading under general linking dynamics in the SIS model. Circles show results of individual-based simulations for the quasi-stationary average fraction of infected 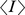 as function of 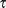. The lower (upper) dashed line shows the analytical prediction of 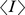 for 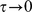 (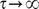), calculated as the average of the quasi-stationary distribution. The analytical prediction in the fast linking limit (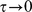) remains valid as long as 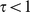, the prediction in the limit of static networks (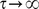) as long as 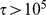. Crosses indicate the quasi-stationary average degree 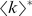 observed in individual-based simulations for given 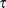. The solid line depicts the analytical prediction of 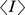 in static networks whose average degree equals the value of 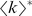 for given 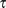. Results in figure illustrate that for 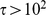, the network dynamics influences disease progression only by controlling 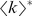. We use 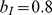, 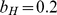, 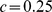, 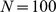 and 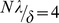.

Up to now we have assumed that the network dynamics proceeds much faster than disease spreading. This may not always be the case, and hence it is important to assess the domain of validity of this limit. In the following, we discuss the particular case of the **SIS** model. An analogous study for both **SIR** and **SI** yields qualitatively similar results, as discussed in the Text S1. Figure 3 shows the average 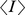 of the quasi-stationary distributions (circles) obtained via computer simulations (see Methods for details) as a function of the relative time scale 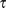 of network update (taking as unit time scale that associated with disease dynamics). Whenever 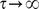, we can characterize the disease dynamics analytically, assuming a well-mixed population (complete graph), whereas for 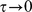 we recover the analytical results obtained in the fast linking limit. At intermediate time scales, Figure 3 shows that as long as 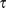 is smaller than 10, network dynamics contributes to inhibit disease spreading by effectively increasing the critical spreading rate. Overall, the validity of the time scale separation extends well beyond the limits one might anticipate based solely on the time separation ansatz. As long as the time scale for network update is smaller than the one for disease spreading (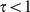), the analytical prediction for the limit 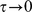, indicated by the lower dashed line in Figure 3, remains valid. The analytical result in the extreme opposite limit (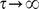), indicated by the upper dashed line in Figure 3, holds as long as 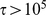. Moreover, it is noteworthy that the network dynamics influences the disease dynamics both by *reducing the frequency of interactions between susceptible and infected*, but also by *reducing the average degree of the network*. These complementary effects are disentangled for intermediate regimes, in which the network dynamics is too slow to warrant sustained protection of susceptible individuals from contacts with infected, despite managing to reduce the average degree (shown by crosses). In fact, for 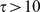 the disease dynamics is mostly controlled by the average degree, as shown by the solid lines in Figure 3. Here, the average stationary distribution was determined by replacing, in the analytic expression for static networks, 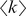 by the time-dependent average connectivity 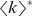 computed numerically.

Note that the specific behavior of 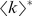 shown in Figure 3 results from the frequency dependence of 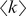. When 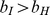, the network will reshape into a configuration with smaller 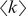 as soon as the disease expansion occurs. For 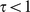, 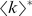 reflects the lifetime of *SS* links, as there are hardly any infected in the population. For 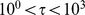, the network dynamics proceeds fast enough to reduce 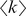, but too slowly to reach its full potential in hindering disease progression. Given the higher fraction of infected, and the fact that *SI* and *II* links have a shorter lifetime than SS links, the average degree drops when increasing 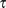 from 1 to 10^3^. Any further increase in 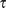 leads to a higher average degree, as the network approaches its static limit.

Contrary to the deterministic **SIS** model, the stochastic nature of disease spreading in finite populations renders certain the probability that the disease disappears after some time. However, this result is of little relevance given the associated times required to reach the absorbing state (except, possibly, in very small communities). Indeed, the characteristic time scale of the dynamics plays a determinant role in the overall epidemiological process and constitutes a central issue in disease spreading.

**Figure 4:**
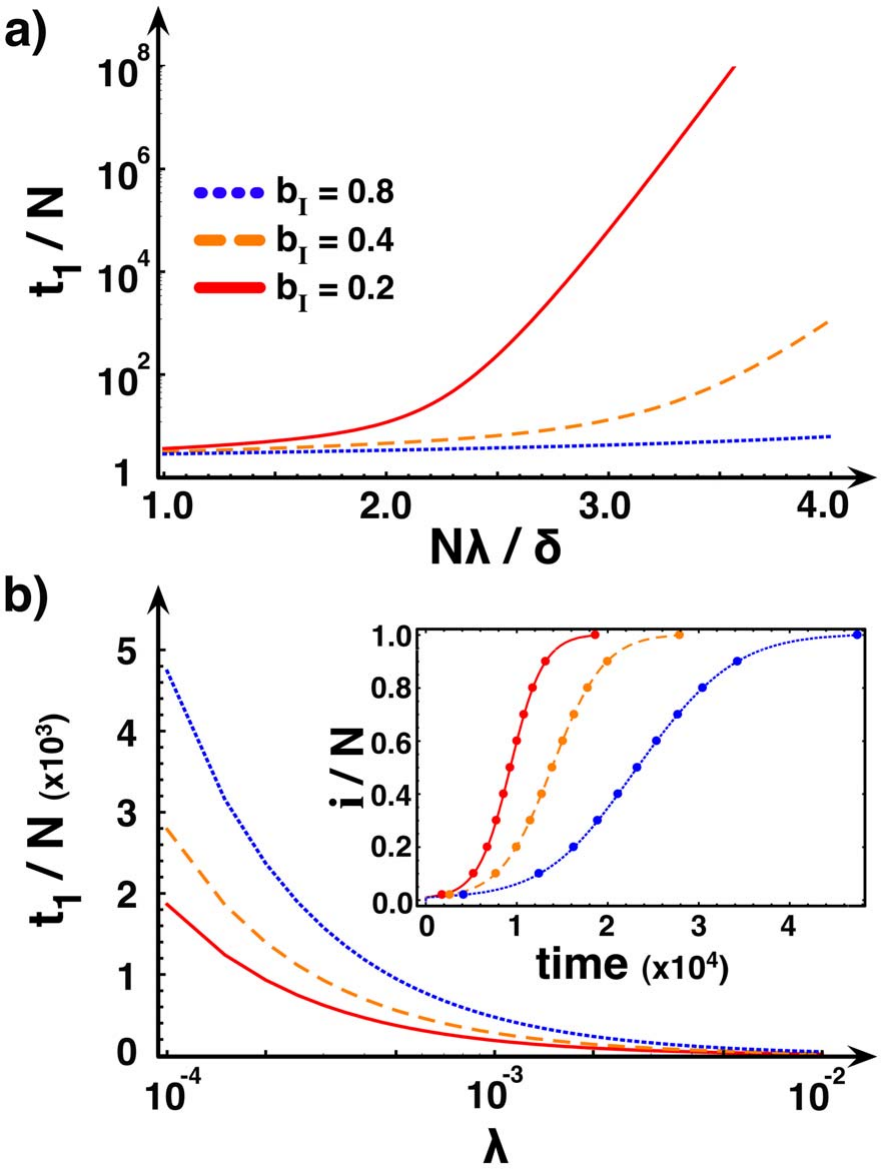
Impact of information on recovery and infection times. **A**) Average number of generations required for disease eradication in an adaptive contact network for different rates *b_I_*, using the **SIS** model. The remaining parameters are 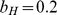, 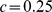 and 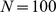. The availability of information drastically reduces the time for disease eradication. **B**) The main plot shows the average number of generations after which a disease infects the entire population in the **SI** model, using the same parameters as in the upper panel. The inset shows how, starting from one infected individual, the fraction of infected changes in time for the same rates *b_I_* and 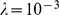. The results obtained via individual-based computer simulations (circles, 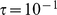) fit perfectly with those calculated analytically (lines).

In the Text S1 we derive an analytical expression for the average recovery time (*t_i_*) of a population with *i* infected individuals. Figure 4A shows the recovery time *t_1_* in adaptive networks for different levels of information, illustrating the spectacular effect brought about by the network dynamics on the recovery time. While on networks without information (*b_I_* = *b_H_*) the recovery time rapidly increases with the rate of infection λ, adding information moves the fraction of infected individuals rapidly to the absorbing state, and, therefore, to the disappearance of the disease. Moreover, as shown in the Text S1, the size of the population can have a profound effect on the recovery times. With increasing population size, the population spends most of the time in the vicinity of the state associated with the interior root of *G(i)*. For large populations, this acts to reduce the intrinsic stochasticity of the dynamics, dictating a very slow extinction of the disease.

When recovery from the disease is impossible, a situation captured by the **SI** model, the population will never become disease-free again once it acquires at least one infected individual. The Markov chain that represents such diseases therefore has another absorbing state, corresponding to networks where everyone has been infected, besides the previous one in which no one is infected. The time to reach this state again depends on the presence of information. When information prevails, susceptible individuals manage to resist infection for a long time, thereby delaying the rapid progression of the disease, as shown in the inset of Figure 4B. Naturally, the average number of generations needed to reach a fully infected population increases with the availability of information, as illustrated in the main panel of Figure 4B.

Finally, in all models discussed here we also investigated the effect of allowing for different individual rates associated with the way each individual creates or destroys her social ties. Due to age-structure of most populations or intrinsic individual or cultural differences, some individuals will tend to react differently whenever they, or a contact, get infected [36], [38]. In the Text S1 we show that the disease spreading remains unaffected when individual rates (of seeking and removing links) are drawn from a normal distribution with variable variance, as long as the average value for the rate remains unchanged.

## Discussion

Making use of three standard models of epidemics involving a finite population in which infection takes place along the links of a dynamical graph, the nodes of which are occupied by individuals, we have shown analytically that the bias in graph dynamics resulting from the availability of information about the health status of others in the population induces fundamental changes in the overall dynamics of disease progression.

The network dynamics proposed here differs from those used in previous models of disease spreading on adaptive networks [21]–[31]. Similar to most models, the population size remains fixed. Unlike most of these studies, however, we do not impose any local or global linking constraints, meaning that individuals can create (or remove) a link without the need for removing (or creating) another one – in other words, the number of links will change in time, also adapting to the disease configuration of the population. Consequently, the average degree of the network results from the self-organization of the network structure, and co-evolves with the disease dynamics (cf. networks evolving towards critical values of connectivity, as studied in [45]). A population suffering from high disease prevalence where individuals avoid contact in order to escape infection will therefore exhibit a lower average degree than a population with hardly any infected individuals. Such a frequency-dependent average degree prevents also that containment of infected individuals would result automatically in the formation of a dense healthy cluster, which is extremely vulnerable to future infection, as reported before in [21], [25], [26].

The description of disease spreading as a stochastic contact process embedded in a Markov chain constitutes a second important distinction between the present model and previous studies. This approach allows for a direct comparison between analytical predictions and individual-based computer simulations, and for a detailed analysis of finite-size effects and convergence times, whose exponential growth will signal possible bistable disease scenarios. In such a framework, we were able to show that adaptive networks in which individuals may be informed about the health status of others lead to a disease whose effective infectiousness depends on the overall number of infected in the population. In other words, disease propagation on adaptive networks can be seen as mathematically equivalent to disease spreading on a well-mixed population, but with a rescaled effective infectiousness. In accord with the intuition advanced in the introduction, as long as individuals react promptly and consistently to accurate available information on whether their acquaintances are infected or not, network dynamics effectively weakens the disease burden the population suffers from. Last but not least, if disease recovery is possible, the time for disease eradication drastically reduces whenever individuals have access to accurate information about the health state of their acquaintances and use it to avoid contact with those infected. If recovery or immunity is impossible, the average time needed for a disease to spread increases significantly when such information is being used. In both cases, our model clearly shows how availability of information hinders disease progression (by means of quick action on infected, e.g., their containment via link removal), which constitutes a crucial factor to control the development of global pandemics.

Finally, it is also worth mentioning that the knowledge about the health state of the others may not always be accurate or available in time. This is for instance the case for diseases where recently infected individuals remain asymptomatic for a substantial period. The longer the incubation period associated with the disease, the less successful individuals will be in escaping infection, which reduces in our model to a lower effective rate of breaking SI links, with the above mentioned consequences. Moreover, the (social) network through which awareness of the health status of others proceeds may lead to different rates of information spread. In such cases, one may model explicitly the spread of the health state of each individual, as done in Refs. [28], [32], and study the interplay between disease expansion and individuals' awareness of the disease. Of course, depending on the particular disease at hand and the contact network along which it propagates, one may have to take other factors into account, besides network adaptability, in order to make accurate predictions of disease progression. Creation and destruction of links may for instance not always occur randomly, as we assumed here, but in a way that is biased by a variety of factors such as social and genetic distance, geographical proximity, family ties, etc. The resulting contact network may therefore become organized in a specific way, promoting the formation of particular structures, such as networks characterized by long-tailed degree distributions or with strong topological correlations among nodes [2], [46]–[48] which, in turn, may influence the disease dynamics. The impact of combining such effects, resulting from specific disease scenarios, with those reported here will depend on their prevalence. A small fraction of non-random links, or of ties which cannot be broken, will likely induce small modifications on the average connectivity of the contact network, which can be incorporated in our analytic expressions without compromising their validity regarding population wide dynamics. On the other hand, when the contact network is highly heterogeneous (e.g., exhibiting pervasive long-tail degree distributions), non-random events may have very distinct effects, from being almost irrelevant (and hence can be ignored) to inducing hierarchical cascades of infection [49], in which case our results will not apply.

## Methods

### Epidemic spreading in finite, well-mixed populations

We define disease dynamics in finite populations of size *N* as a stochastic process. Time evolves in discrete steps and two types of events may occur which change the composition of the population: *infection* events and *recovery* events. Let us assume the **SIR** model, as this is the most general case (see Text S1 for a detailed analysis specific for each disease model). Each state of the population is characterized by two indices 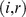, where *i* is the number of infected individuals in the population and *r* the number of recovered (and immune) individuals (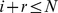). The number of infected decreases with a rate given by
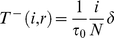
(M1)where 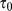 is the recovery time scale, 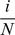 the probability that a randomly selected individual is infected and 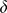 the probability that this individual recovers.

Adopting 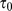 as a reference, we assume that the higher the average number of contacts 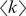, the smaller the time scale 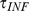 at which infection update events occur (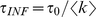) [3]. Hence, the rate of increasing the number of infected is given by
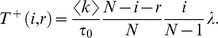
(M2)The first factor stands for the typical time scale at which infection events occur. The second factor represents the probability of randomly picking a healthy individual. This individual interacts with a random neighbor, who is infected with probability given by the third factor (well-mixed assumption). This contact leads to an additional infection with probability 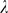. When 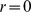, Equations (M1) and (M2) describe both **SIS** and **SI** (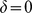) models.

We obtain the finite population analogue of the well-known mean-field equations characteristic of these models by recognizing that, in the limit of large populations, 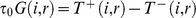
provides the rate of change of infected individuals. For large *N*, replacing 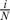 by *x* and 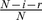 by *y*, the *gradients of infection* are given by
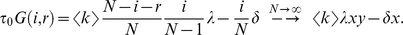
(M3)Assuming a fixed number of recovered individuals 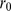, 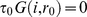 for 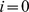 and 
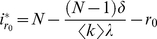
. Moreover, 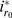 becomes the finite population equivalent of an interior equilibrium for 
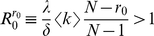
. The disease will most likely expand when 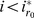, the opposite happening otherwise. The same condition holds for the **SIS** model, taking 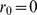.

### Quasi-stationary distributions in finite populations

Equations (M1) and (M2) define a Markov chain *M* with variable states depending on the epidemic model (see Text S1). The fraction of time the population spends in each state is given by the stationary distribution of *M*, which is defined as the eigenvector associated with eigenvalue 1 of the transition matrix of *M* [50], [51]. In the **SIS** model, the state without infected (*i* = 0) is, however, an absorbing state of the Markov Chain. The quantity of interest is therefore the stationary distribution of the Markov chain obtained from *M* by excluding the absorbing state *i = 0*. This distribution is also known as the quasi-stationary distribution of *M* [52] and corresponds to the fraction of time the population spends in each state, assuming the disease does not go extinct (see Figure 1).

### Network dynamics

Consider a network of constant size *N* with variable number of links. New links are established randomly at rate *c*. Existing links between individuals with health states *p* and *q* (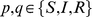) disappear at rate *b_pq_*. The time evolution of the number *L_pq_* of *pq*-links can be written as a system of ordinary differential equations 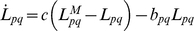 [39], [40]. 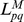 denotes the maximum number of *pq*-links, which depends on the number of individuals in states *p* and *q* (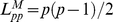 and 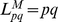 for 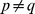) and thereby couples the network dynamics to the disease dynamics. In the steady state of the linking dynamics (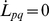), the number of links of links of each type is given by 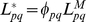, with 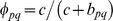.

### Epidemic spreading in dynamical networks

When the time scale for network update (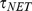) is much smaller than the one for disease spreading (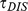), the number of infected increases with a rate
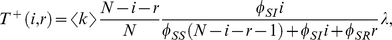
(M4)where we made 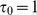. The effect of the network dynamics becomes apparent in the third factor, which represents the probability that a randomly selected neighbor of a susceptible is infected. In addition, Equation (M1) remains valid, as the linking dynamics does not affect the rate at which the number of infected decreases. Similarly to the case of static networks, one can show that the disease remains endemic in the **SIS** model whenever 
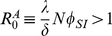
. A similar result holds for the **SIR** model (see Text S1). It is noteworthy that we can write Equation M4 as follows
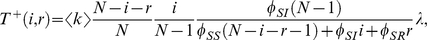
(M5)which shows that disease spreading in an adaptive network is equivalent to that in a well-mixed population with frequency dependent average degree 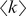 and a transmission probability that is rescaled according to 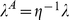, where
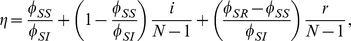
(M6)which is valid for both **SIR**, **SIS** (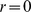) and **SI** (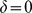,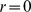) models.

### Computer simulations

All individual-based simulations start from a complete network of size *N* = 100. Disease spreading and network evolution proceed together under asynchronous updating. Disease update events take place with probability 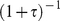, where 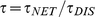 and 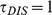, network update events occur otherwise. For network update events, we randomly draw two nodes from the population. If connected, then the link disappears with probability given by the respective *b_pq_*. Otherwise, a new link appears with probability *c*. When a disease update event occurs, a recovery event takes place with probability 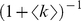, an infection event otherwise. In both cases, an individual *j* is drawn randomly from the population. If *j* is infected and a recovery event has been selected then *j* will become susceptible (or recovered, model dependent) with probability δ. If *j* is susceptible and an infection event occurs, then *j* will get infected with probability λ if a randomly chosen neighbor of *j* is infected. The quasi-stationary distributions shown in Figure 1 are computed as the fraction of time the population spends in each configuration (i.e., number of infected individuals) during 10^9^ disease event updates (10^7^ generations). The average number of infected 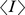 and the mean average degree of the network 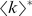 observed during these 10^7^ generations are shown in Figure 3. The results reported are independent of the initial number of infected in the network. Finally, the disease progression in time, shown in Figure 4b, is calculated from 10^4^ independent simulations, each simulation starting with 1 infected individual. The reported results correspond to the average amount of time after which the population reaches a state with *i* infected.

## Supporting Information

Text S1 Adaptive contact networks change effective disease infectiousness and dynamics. 1. The SIS model. 1.1 Recovery times in finite populations. 2. The SI model. 2.1. Infection times in finite populations. 2.2. Infection times in dynamical networks. 3. The SIR model. 3.1. The SIR model in finite populations. 3.2. The SIR model in dynamical networks. 4. Individual diversity in linking dynamics.
(2.08 MB PDF)
