# Supplementary material for: Adaptive Contact Networks Change Effective Disease Infectiousness and Dynamics
Source: PLoS Comput Biol. 2010 Aug 19;6(8):e1000895. doi: 10.1371/journal.pcbi.1000895 (PMC2924249; doi:10.1371/journal.pcbi.1000895)
Supplement: Text S1 — Adaptive contact networks change effective disease infectiousness and dynamics. 1. The SIS model. 1.1 Recovery times in finite populations. 2. The SI model. 2.1. Infection times in finite populations. 2.2. Infection times in dynamical networks. 3. The SIR model. 3.1. The SIR model in finite populations. 3.2. The SIR model in dynamical networks. 4. Individual diversity in linking dynamics. (2.08 MB PDF) [file pcbi.1000895.s001.pdf]

TEXT S1

Adaptive contact networks change effective disease infectiousness and dynamics

Sven Van Segbroeck[1,2,6], Francisco C. Santos[3,5,6] and Jorge M. Pacheco[4,5,6]

[1] COMO, Vrije Universiteit Brussel, Pleinlaan 2, 1050 Brussels, Belgium.
[2] MLG, Département d'Informatique, Université Libre de Bruxelles, Boulevard du Triomphe CP212, 1050 Brussels, Belgium.
[3]CENTRIA, Departamento de Informática, Faculdade de Ciências e Tecnologia, Universidade Nova de Lisboa, 2829-516 Caparica, Portugal.
[4] Departamento de Matemática e Aplicações, Universidade do Minho, 4710 - 057 Braga, Portugal
[5] ATP-group, CMAF, Complexo Interdisciplinar, P-1649-003 Lisboa Codex, Portugal,
[6] GADGET, Apartado 1329, 1009-001, Lisboa, Portugal.

Supporting information summary:

1 supporting text with
- **17** pages (including title page)
- **17974** characters (including spaces)
- **4** Figures

Overview:

1. The **SIS** model
   1.1 Recovery times in finite populations

2. The **SI** model
   2.1. Infection times in finite populations
   2.2. Infection times in dynamical networks

3. The **SIR** model
   3.1. The **SIR** model in finite populations
   3.2. The **SIR** model in dynamical networks

4. Individual diversity in linking dynamics

1. The SIS model

In the **SIS** model individuals can be in one of two epidemiological states: Infected (*I*) or Susceptible (*S*). Each disease is characterized by a recovery rate (δ) and an infection contact rate (λ). In an infinite, well-mixed population, the fraction of infected individuals (*x*) changes in time according to the following differential equation

$$\dot{x} = \lambda\langle k\rangle xy - \delta x$$

where $y = 1 - x$ is the fraction of susceptible individuals and $\langle k\rangle$ the average number of contacts of each individual [1]. There are two possible *equilibria* ($\dot{x} = 0$): $x = 0$ and $x = 1 - R_0^{-1}$, where $R_0 = \lambda\langle k\rangle / \delta$ denotes the basic reproductive ratio. The value of R_0 determines the stability of these two equilibria: $x = 1 - R_0^{-1}$ is stable when $R_0 > 1$ and unstable when $R_0 < 1$.

We defined a discrete stochastic process describing the disease dynamics associated with the **SIS** model in a finite population (see Methods) which can be represented as a Markov chain *M* with *N+1* states [2,3], illustrated in the following figure:

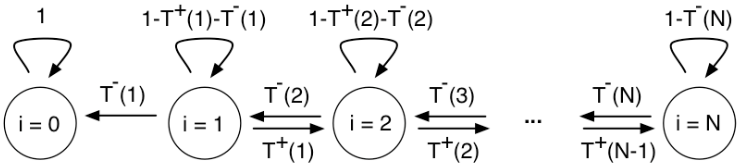

Each configuration of the population, which is defined by the number of infected individuals *i*, corresponds to one state of the Markov chain. In a finite, well-mixed population, Equations M1 and M2 (with *r*=0) define the transitions between different states. In a dynamical contact network, Equations M1 and M4 define those transitions (assuming that the linking dynamics proceeds much faster than the disease dynamics). This way, we obtain the following transition matrix for *M*:

$$P = \begin{bmatrix} 1 & 0 & 0 & \dots & 0 & 0 & 0 \\ T_1^- & 1-T_1^+-T_1^- & T_1^+ & \dots & 0 & 0 & 0 \\ \vdots & \vdots & \vdots & \ddots & \vdots & \vdots & \vdots \\ 0 & 0 & 0 & \dots & T_{N-1}^- & 1-T_{N-1}^+-T_{N-1}^- & T_{N-1}^+ \\ 0 & 0 & 0 & \dots & 0 & T_N^- & 1-T_N^- \end{bmatrix} \quad (1)$$

where each element p_{kj} of P represents the probability of moving from state k to state j during one time step. The state without any infected individual (i=0) is an absorbing state of the Markov chain. In other words, the disease always dies out and once this happens, it never re-appears.

1.1 Recovery times in finite populations

Here, we derive an analytical expression for the average time it takes to reach the single absorbing state of the **SIS** Markov chain (i.e., the average *recovery time*). Let us denote by $P_i(t)$ the probability that the disease disappears at time t when starting with i infected individuals at time *0*. Hence, the average recovery time is given by [4]

$$t_i \equiv \sum_{t=0}^{\infty} t P_i(t). \quad (2)$$

Equation 1 yields the following recurrence relation for $P_i(t)$:

$$\begin{aligned} &P_0(t) = \delta_{t,0} \\ &P_i(t) = T_i^- P_{i-1}(t-1) + \left(1 - T_i^+ - T_i^-\right) P_i(t-1) + T_i^+ P_{i+1}(t-1) \\ &P_N(t) = T_N^- P_{N-1}(t-1) + \left(1 - T_N^-\right) P_N(t-1). \end{aligned} \quad (3)$$

We multiply both sides of these equations by t and sum from $t = 0$ to ∞

$$\begin{aligned} &\sum_{t=0}^{\infty} t P_i(t) = T_i^- \sum_{t=0}^{\infty} t P_{i-1}(t-1) + \left(1 - T_i^+ - T_i^-\right) \sum_{t=0}^{\infty} t P_i(t-1) + T_i^+ \sum_{t=0}^{\infty} t P_{i+1}(t-1) \\ &\sum_{t=0}^{\infty} t P_N(t) = T_N^- \sum_{t=0}^{\infty} t P_{N-1}(t-1) + \left(1 - T_N^-\right) \sum_{t=0}^{\infty} t P_N(t-1). \end{aligned} \quad (4)$$

Since M has only one absorbing state, we know that $\sum_{t=0}^{\infty} P_i(t) = 1$ for all values of i. The sums on the right hand side of Equation 4 can therefore be written in terms of the average recovery time t_i as follows

$$\begin{aligned}
&\sum_{t=0}^{\infty} tP_{i-1}(t-1) = \sum_{t=0}^{\infty} (t+1)P_{i-1}(t) = t_{i-1}+1 \\
&\sum_{t=0}^{\infty} tP_{i}(t-1) = \sum_{t=0}^{\infty} (t+1)P_{i}(t) = t_{i}+1 \\
&\sum_{t=0}^{\infty} tP_{i+1}(t-1) = \sum_{t=0}^{\infty} (t+1)P_{i+1}(t) = t_{i+1}+1,
\end{aligned} \tag{5}$$

which leads to the following recurrence relation for t_i

$$t_i = T_i^- t_{i-1} + \left(1 - T_i^- - T_i^+\right) t_i + T_i^+ t_{i+1} + 1, \tag{6}$$

and for t_N

$$t_N = T_N^- t_{N-1} + \left(1 - T_N^-\right) t_N + 1. \tag{7}$$

We introduce a variable $s_i \equiv t_i - t_{i-1} (i = 1, \ldots, N)$ for which we can derive the following recurrence relation by using Equation 6

$$\begin{aligned}
&T_i^+ t_{i+1} - T_i^+ t_i = T_i^- t_i - T_i^- t_{i-1} - 1 \\
&\Leftrightarrow T_i^+ s_{i+1} = T_i^- s_i - 1 \\
&\Leftrightarrow s_{i+1} = \frac{T_i^-}{T_i^+} s_i - \frac{1}{T_i^+} .
\end{aligned} \tag{8}$$

Note that this equation is valid for all $i=1,\ldots,N-1$. Equation 7, on the other hand, can be written as $T_N^- t_N - T_N^- t_{N-1} = 1$ so that

$$s_N = \frac{1}{T_N^-} . \tag{9}$$

In the following, we use auxiliary variables $\gamma_i = \frac{T_i^-}{T_i^+}$ and $q_i = \prod_{l=1}^{i} \gamma_l$. Equation 8 allows us to write s_i $(i=2,\ldots,N)$ as a function of t_1

$$\begin{aligned}
s_i &= \frac{T_{i-1}^-}{T_{i-1}^+} s_{i-1} - \frac{1}{T_{i-1}^+} \\
&= \frac{T_{i-1}^-}{T_{i-1}^+}\frac{T_{i-2}^-}{T_{i-2}^+} s_{i-2} - \frac{T_{i-1}^-}{T_{i-1}^+}\frac{1}{T_{i-2}^+} - \frac{1}{T_{i-1}^+} = \frac{T_{i-1}^-}{T_{i-1}^+}\frac{T_{i-2}^-}{T_{i-2}^+}\frac{T_{i-3}^-}{T_{i-3}^+} s_{i-3} - \frac{T_{i-1}^-}{T_{i-1}^+}\frac{T_{i-2}^-}{T_{i-2}^+}\frac{1}{T_{i-3}^+} \\
&= \gamma_{i-1}\gamma_{i-2}\gamma_{i-3}s_{i-3} - \gamma_{i-1}\gamma_{i-2}\frac{1}{T_{i-3}^+} - \gamma_{i-1}\frac{1}{T_{i-2}^+} - \frac{1}{T_{i-1}^+} \\
&= \left(\prod_{l=1}^{i-1}\gamma_l\right)s_1 - \left(\prod_{l=1}^{i-1}\gamma_l\right)\left(\sum_{k=1}^{i-1}\frac{1}{T_k^+\prod_{j=1}^{k}\gamma_j}\right) = q_{i-1}s_1 - q_{i-1}\sum_{k=1}^{i-1}\frac{1}{T_k^+ q_k},
\end{aligned}$$

and therefore

$$s_i = q_{i-1}t_1 - q_{i-1}\sum_{k=1}^{i-1}\frac{1}{T_k^+ q_k}. \tag{10}$$

Combining Equations 9 and 10 leads to the following expression for t_1

$$t_1 = \frac{1}{q_{N-1}T_N^-} + \sum_{k=1}^{N-1}\frac{1}{T_k^+ q_k}, \tag{11}$$

such that t_i can be written as a function of t_1 as follows

$$t_i = \sum_{k=1}^{i} s_k = t_1\sum_{k=0}^{i-1} q_k - \sum_{k=0}^{i-1} q_k \sum_{k=0}^{i-1}\frac{1}{T_j^+ q_j}. \tag{12}$$

The intrinsic stochasticity of the model, resulting from the finiteness of the population, makes the disease disappear from the population after a certain amount of time. As such, the population size plays an important role in the average recovery time associated with a certain disease, a feature we discuss in this section. We consider static networks of different size *N*, keeping the average degree $\langle k \rangle$ fixed, and plot the average time to disease eradication t_1 as a function of $\langle k \rangle \lambda / \delta$ (see upper panel of Figure 1). Whenever $\langle k \rangle \lambda / \delta > 1$, the existence of an interior root in *G(i)* leads to a dramatic increase of the recovery time (note the logarithmic scale) to extremely high values compared with those obtained for low infection rates. This problem becomes particularly acute in large populations because the fraction of time the population

spends in each state is not the same for different population sizes. The lower panel of Figure 1 shows the fingerprint of the population size on the quasi-stationary distribution for static contact networks with fixed average degree $\langle k \rangle = 49$, for $\langle k \rangle \lambda / \delta = 2$. With increasing population size, the population spends less and less time close to the single absorbing state of the dynamics, remaining instead in the vicinity of the state associated with the interior root of *G(i)*. This acts to reduce the intrinsic stochasticity of the dynamics, dictating a very slow convergence towards the absorbing state (no disease).

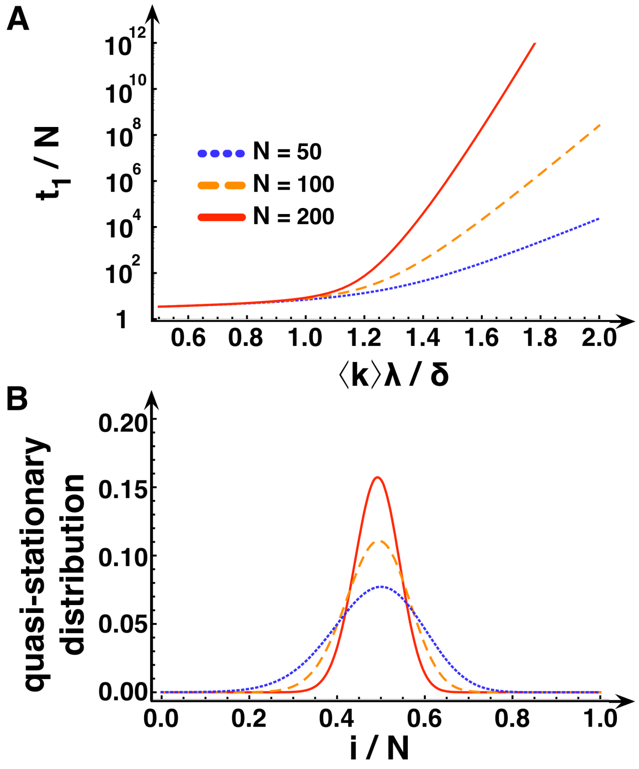

Figure 1. Impact of population size on recovery times. A) Average number of generations required for disease eradication in the SIS model in static networks of different size *N*, while keeping the average degree $\langle k \rangle$ constant (equal to 49). B) Quasi-stationary distribution of the number of infected for the same values of *N* and $\langle k \rangle$. The disease parameters satisfy $\langle k \rangle \lambda / \delta = 2$.

2. The SI model

The **SI** model is mathematically equivalent to the **SIS** model with $\delta = 0$, and has been employed to study for instance the dynamics of AIDS. The Markov Chain representing the disease dynamics is therefore defined by transition matrix Equation 1, with $T_i^- = 0$ for all i. The remaining transition probabilities T_i^+ $(0 < i < N)$ are exactly the same as for the **SIS** model. Disease spreading in an adaptive contact network will therefore be equivalent again to that in a well-mixed population with a transmission probability that is rescaled according to Equation 6.

Since all T_i^- equal zero, the Markov Chain has two absorbing states: the canonical one without any infected (i=0) and the one without any susceptible (i=N). The disease will expand continuously as soon as one individual in the population gets infected, ultimately leading to a fully infected population. The average amount of time after which this happens, which we refer to as the *average infection time*, constitutes the main quantity of interest and can be calculated analytically, as discussed below.

2.1. Infection times in finite populations

Let us denote by $P_i(t)$ the probability to reach 100% of infected at time t, when starting with i infected individuals at time 0. The average infection time is then given by

$$t_i \equiv \sum_{t=0}^{\infty} t P_i(t). \tag{13}$$

The transition matrix of the Markov Chain yields the following recurrence relation for $P_i(t)$:

$$P_0(t) = 0$$
$$P_i(t) = \left(1 - T_i^+\right)P_i\left(t-1\right) + T_i^+ P_{i+1}\left(t-1\right) \qquad (13)$$
$$P_N(t) = \delta_{t,0}.$$

Multiplying both sides of the equation by t and summing from $t = 0$ to ∞ results in the following expression for t_i:

$$t_i = (1 - T_i^+)\sum_{t=0}^{\infty} tP_i\left(t-1\right) + T_i^+ \sum_{t=0}^{\infty} tP_{i+1}\left(t-1\right). \qquad (14)$$

Since $\sum_{t=0}^{\infty} P_i(t) = 1$, for all $i \neq 0$, we can write the sums at the right hand side of Equation 14 in terms of t_i

$$\sum_{t=0}^{\infty} tP_i(t-1) = \sum_{t=0}^{\infty}\left(t+1\right)P_i(t) = t_i + 1$$
$$\sum_{t=0}^{\infty} tP_{i+1}(t-1) = \sum_{t=0}^{\infty}\left(t+1\right)P_{i+1}(t) = t_{i+1} + 1,$$

and obtain the following recurrence relation for t_i

$$t_i = \left(1 - T_i^+\right)t_i + T_i^+ t_{i+1} + 1, \qquad (15)$$

which reduces to

$$t_i = t_{i-1} - \frac{1}{T_{i-1}^+} = t_1 - \sum_{j=1}^{i-1}\frac{1}{T_j^+}. \qquad (16)$$

Since $t_N = 0$, Equation 15 for $i = N - 1$ reduces to

$$t_{N-1} = \left(1 - T_{N-1}^+\right)t_{N-1} + 1, \qquad (17)$$

so that $t_{N-1} = \frac{1}{T_{N-1}^+}$. By combining this with Equation 16, we obtain the following expression for t_1:

$$t_1 = \sum_{j=1}^{N-1}\frac{1}{T_j^+}. \qquad (18)$$

Hence, the average number of time steps needed to reach 100% infection, starting from i infected equals

$$t_i = \sum_{j=i}^{N-1} \frac{1}{T_j^+}. \tag{19}$$

2.2. Infection times in dynamical networks

The main text discusses how the availability of information regarding the health status of one's partners affects the average infection time. Here, we use computer simulations to verify to which extent these results, obtained analytically via time scale separation, remain valid for intermediate values of the relative timescale τ for the linking dynamics. We start with a complete network of size N, in which initially one individual is infected, the rest being susceptible. Disease spreading and network evolution proceed simultaneously under asynchronous updating. Network update events take place with probability $(1+\tau)^{-1}$, **SI** state update events occur otherwise. Both processes are defined in exactly the same way as in the **SIS** model, taking $\delta = 0$ (see Methods). For each value of τ, we run 10^4 simulations and calculate the average number of generations after which the population becomes completely infected. These values are depicted in Figure 2. The lower dashed line indicates the analytical prediction of the infection time in the limit $\tau \to \infty$, which we already recover in simulation for $\tau > 10^2$. When τ is smaller than 10^2, the average infection time significantly increases, and already reaches the analytical prediction for the limit $\tau \to 0$ (indicated by the upper dashed line) when $\tau < 1$. Hence, the validity of the time scale separation does again extend well beyond the limits one might expect.

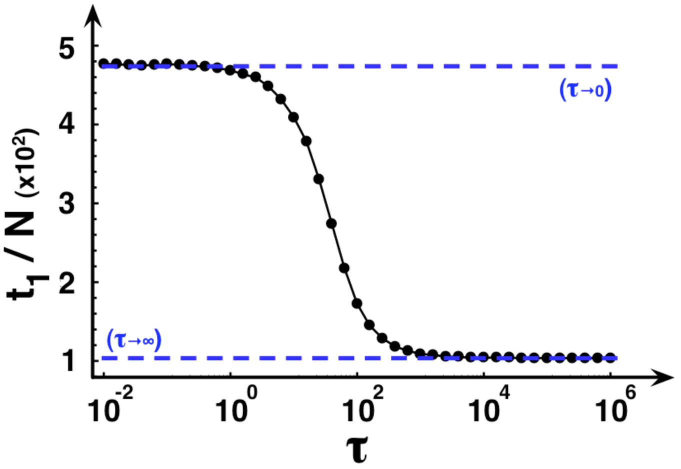

Figure 2. Disease spreading in the SI model for variable time scales τ of the linking dynamics. Solid circles show the average number of generations to reach a fully infected population, starting from one single infected individual, obtained in simulation. Dashed lines indicate the analytical predictions for these values, either in the limit $\tau \to 0$ (upper dashed line), or in the limit $\tau \to \infty$ (lower dashed line). We use the following parameter values: $b_I = 0.8$, $b_H = 0.2$, $c = 0.25$, $N = 100$ and $\lambda = 0.001$.

3. The SIR model

With **SIR** one models diseases in which individuals acquire immunity after recovering from infection. Typical examples of situations potentially modeled with **SIR** are flu over a single season or computer virus epidemics. We distinguish three epidemiological states to model the dynamics of such diseases: susceptible (S), infected (I) and, finally, recovered (R), indicating those who have become immune to further infection.

The **SIR** model in infinite, well-mixed populations is defined by a recovery rate δ and an infection contact rate λ. The fraction of infected individuals x changes in time according to the following differential equation

$$\dot{x} = \langle k \rangle \lambda x y - \delta x \,, \qquad (20)$$

where y denotes the fraction of susceptible individuals, which in turn changes according to

$$\dot{y} = -\langle k \rangle \lambda x y \,. \tag{21}$$

Finally, the fraction of individuals z in the recovered class changes according to

$$\dot{z} = \delta \, x \,. \tag{22}$$

3.1. The SIR model in finite populations

We address the **SIR** model in finite, well-mixed populations in the same way as we addressed the **SIS** and **SI** models. The Markov Chain describing the disease dynamics becomes slightly more complicated and has states (i,r), where i is the number of infected individuals in the population and r the number of recovered (and immune) individuals ($i + r \leq N$). A schematic representation of the Markov Chain is given in the following figure:

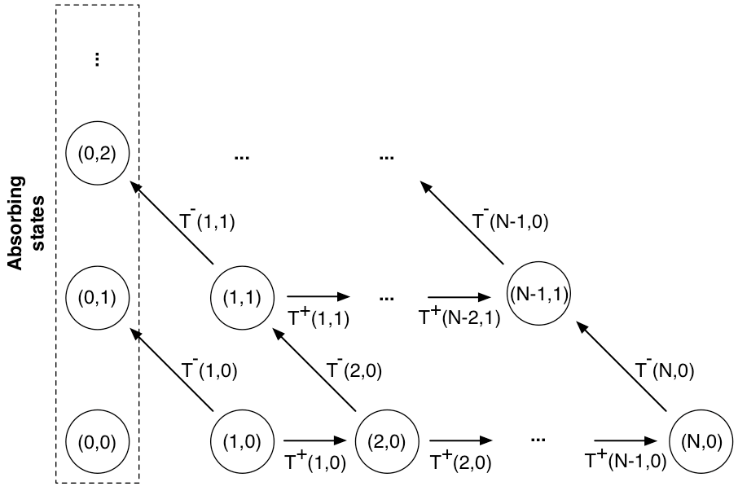

Note that the states $(0,r)$, with $0 \leq r \leq N$, are absorbing states. Each of these states corresponds to the number of individuals that are (or have become) immune at the time the disease goes extinct.

Consider a population of size N with average degree $\langle k \rangle$. The number of infected will increase with a rate

$$T^{+}(i,r) = \frac{\langle k \rangle}{\tau_0} \frac{N-i-r}{N} \frac{i}{N-1} \lambda \qquad (23)$$

and decrease with a rate

$$T^{-}(i,r) = \frac{1}{\tau_0} \frac{i}{N} \delta, \qquad (24)$$

where τ_0 denotes the recovery time scale. The gradient of infection, which measures the likelihood for the disease to either expand or shrink in a given state, is given by

$$\tau_0 G(i,r) = \langle k \rangle \frac{N-i-r}{N} \frac{i}{N-1} \lambda - \frac{i}{N} \delta \xrightarrow{N \to \infty} \langle k \rangle \lambda x y - \delta x. \qquad (25)$$

Note that we recover Equation 20 in the limit $N \to \infty$. For a fixed number of recovered individuals r_0, we have that $\tau_0 G(i,r_0) = 0$ for $i = 0$ and for $i^*_{r_0} = N - \frac{(N-1)\delta}{\langle k \rangle \lambda} - r_0$. For $R_0^{r_0} = \langle k \rangle \frac{\lambda}{\delta} \frac{N-r_0}{N-1} > 1$, $i^*_{r_0}$ becomes the finite population analogue of an interior equilibrium. Furthermore, one can show that the partial derivative $\frac{\partial\, G(i,r)}{\partial\, i}$ has at most one single root in (0,1), possibly located at $\bar{i}_{r_0} = \frac{i^*_{r_0}}{2} \le i^*_{r_0}$. Hence, $G(i,r_0)$ reaches a maximum at $\bar{i}_{r_0}$ ($\frac{\partial^2 G(i,r)}{\partial\, i^2} = -\frac{2\langle k \rangle \lambda}{N(N-1)} < 0$). The number of infected will therefore most likely increase for $i < i^*_{r_0}$ (assuming r_0 immune individuals), and most likely decrease otherwise.

The gradient of infection determines the probability to end up in each of the different absorbing states of the Markov chain. These probabilities can be calculated analytically as follows. Let us use $y^a_{i,r}$ to denote the probability that the population

ends up in the absorbing state with *a* recovered individuals, starting from a state with *i* infected and *r* recovered. We obtain the following recurrence relationship for $y_{i,r}^a$

$$y_{i,r}^a = T^-(i,r)y_{i-1,r+1}^a + T^+(i,r)y_{i+1,r}^a + (1 - T^-(i,r) - T^+(i,r))y_{i,r}^a, \quad (26)$$

which reduces to

$$y_{i,r}^a = \left(T^-(i,r) + T^+(i,r)\right)^{-1}\left(T^-(i,r)y_{i-1,r+1}^a + T^+(i,r)y_{i+1,r}^a\right). \quad (27)$$

The following boundary conditions

$$\begin{aligned} y_{0,r}^r &= 1 \\ y_{0,r}^a &= 0 \; if \;\; r \neq a \\ y_{i,r}^a &= 0 \; if \;\; i + r > a \end{aligned} \quad (28)$$

allow us to compute $y_{i,r}^a$ for every *a*, *i* and *r*.

3.2. The SIR model in dynamical networks

We adopt the same convention as before and normalize the recovery time scale τ_0 to 1. In the fast linking limit, the number of infected increases with a rate given by

$$T^+(i,r) = \langle k \rangle \frac{N - i - r}{N} \frac{\phi_{SI} i}{\phi_{SS}(N - i - r - 1) + \phi_{SI} i + \phi_{SR} r} \lambda \;. \quad (29)$$

The rate with which the number of infected decreases is network independent and equal to

$$T^-(i,r) = \frac{i}{N}\delta. \quad (30)$$

We can write Equation 26 as follows

$$T^+(i,r) = \langle k \rangle \frac{N - i - r}{N} \frac{i}{N-1} \frac{\phi_{SI}(N-1)}{\phi_{SS}(N - i - r - 1) + \phi_{SI} i + \phi_{SR} r} \lambda, \quad (31)$$

so that the disease dynamics in an adaptive contact network becomes once again *equivalent to* that in a well-mixed population with a transmission probability that is rescaled according to $\lambda^A = \eta^{-1}\lambda$, where

$$\eta = \frac{\phi_{SS}}{\phi_{SI}} + \left(1 - \frac{\phi_{SS}}{\phi_{SI}}\right)\frac{i}{N-1} + \left(\frac{\phi_{SR} - \phi_{SS}}{\phi_{SI}}\right)\frac{r}{N-1}. \quad (32)$$

The gradient of infection allows us to characterize, once more, when disease expansion will either be most likely or least likely. To do so, we study the partial derivative $\frac{\partial G(i,r)}{\partial i}$ at $i = 0$

$$\left.\frac{\partial G(i,r)}{\partial i}\right|_{i=0} = -\frac{\delta}{N} + \frac{N-r}{N^2}\lambda\phi_{SI}\left[2(N-r) + \frac{r(r-1)\phi_{RR} - (N-r)(N-r-1)\phi_{SS}}{r\phi_{SR} + (N-r-1)\phi_{SS}}\right]. \quad (33)$$

This equation exceeds zero whenever

$$\phi_{SI}\frac{\lambda}{\delta}\frac{N-r}{N}\left[2(N-r) + \frac{r(r-1)\phi_{RR} - (N-r)(N-r-1)\phi_{SS}}{r\phi_{SR} + (N-r-1)\phi_{SS}}\right] > 1. \quad (34)$$

Note that taking $r = 0$ yields the basic reproductive ratio R_0^A for both **SIR** and **SIS**:

$$R_0^A \equiv N\phi_{SI}\frac{\lambda}{\delta} > 1. \quad (35)$$

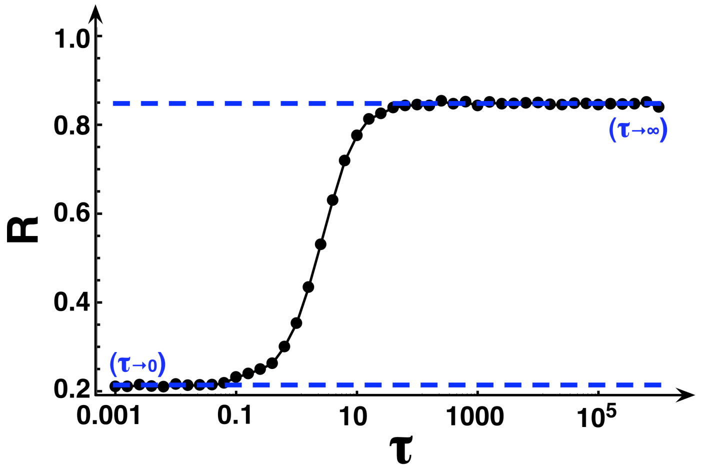

Figure 3. Disease spreading in the SIR model for variable time scales τ of the linking dynamics. Solid circles show the final fraction of recovered individuals as a function of τ in populations with initially one infected. The upper (lower) dashed line shows the corresponding analytical prediction in the limit $\tau \to \infty$ ($\tau \to 0$). We use the following parameter values: $b_I = 0.8$, $b_H = 0.2$, $c = 0.25$, $\lambda = 0.01$, $\delta = 0.15$ and $N = 100$.

Figure 2 of the main text gives a complete picture of the gradient of infection in the fast linking limit, showing how availability of information modifies the spreading dynamics and reduces the number of individuals that will be affected by the disease. In the following, we study to which extent such result remains true for variable time scales τ of the linking dynamics. We start again with a complete network of size *N* in which one single individual is infected, the rest being susceptible. The simulations proceed exactly as before, i.e., network evolution proceeds simultaneously with disease spreading. Network update events take place with probability $(1+\tau)^{-1}$, **SIR** state update events occur otherwise. Each simulation runs until the disease goes extinct. For each value of τ, we run 10^4 simulations and average the final fraction of individuals that have been affected by the disease, which corresponds to the final fraction of individuals in the recovered class. These results are depicted in Figure 3. The upper dashed line indicates the expected fraction of recovered individuals in a static network ($\tau \to \infty$). This value is obtained by calculating $\sum_{i=0}^{N} i\, y_{1,0}^{i}$, where $y_{1,0}^{i}$ is given by Equations 27 and 28. One observes that linking dynamics does not affect disease dynamics for $\tau > 10$. Once τ drops below 10, a significantly smaller fraction of individuals is affected by the disease. This fraction reaches the analytical prediction for $\tau \to 0$ as soon as $\tau < 0.1$. Hence, once again, results obtained via separation of time scales remain valid for a wide range of intermediate time scales.

4. Individual diversity in linking dynamics

So far, we have studied the role of network adaptation on disease dynamics, assuming that individuals can be considered equivalent in all respects, apart from their epidemiological state. However, in realistic human webs of contacts, not all

individuals are equally likely to engage in new interactions with each other, depending on different factors such as social and genetic distance, geographical proximity, family ties, etc [1,5-9]. On the other hand, the very same factors may also influence an individual's capacity of breaking existing links. Hence, generally speaking, some links will last longer (will be created faster) than others, and the question is to which extent such diversity in individual behavior influences disease dynamics. As pointed out in the discussion, there may be situations in which links cannot be set up or removed randomly. Such features, of course, cannot be captured with the present model.

Here, we address individual diversity by means of numerical simulations. Before, we considered a fixed characteristic rate b_{pq} for breaking links of type pq (p and q $q \in \{S,I,R\}$), and another fixed rate c for creating new links. Instead of using these fixed values for all individuals, we now assign different rates to each *pair* of individuals, thereby accounting for a variety of factors that influence the social conditions between different individuals. Specifically, we assign rates c_{ab} and b_{pq}^{ab} to each pair of individuals a and b, their explicit values being drawn from a Gaussian distribution with mean c and b_{pq}, respectively, and standard deviation $\sigma > 0$.

Figure 4 shows the fraction of recovered/immune individuals after disease extinction for the **SIR** model, but similar conclusions are obtained for the **SI** and **SIS** models (not shown). Our results show that diversity in the way individuals create and remove links (which may originate from a broad range of factors) does not affect the overall final fraction of individuals in the recovered class, as long as the average rates remain unchanged. In summary, to the extent that individual diversity can be recast in the form modeled here, our results discussed in the main text remain valid.

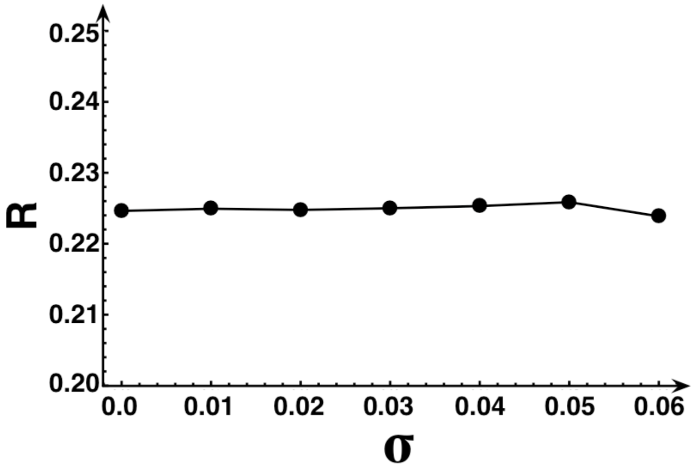

Figure 4. Fraction of recovered individuals after disease extinction for the SIR model as a function of the standard deviation σ associated with the normal distribution from which the rates that define the linking dynamics are drawn. We use the following parameter values: $b_I = 0.8$, $b_H = 0.2$, $c = 0.25$, $\lambda = 0.01$, $\delta = 0.15$, $\tau = 0.1$ and $N = 100$.

References

1. Barrat A, Barthelemy M, Vespignani A (2008) Dynamical processes in complex networks. Cambridge: Cambridge University Press.
2. Karlin S, Taylor HE (1975) A First Course in Stochastic Processes: Academic Press.
3. Van Kampen NG (2007) Stochastic Processes in Physics and Chemistry: North Holland.
4. Antal T, Scheuring I (2006) Fixation of Strategies for an Evolutionary Game in Finite Populations. Bull Math Biol 68: 1923-1944.
5. Anderson RM, May RM (1992) Infectious Diseases in Humans. Oxford: Oxford University Press.
6. Colizza V, Barrat A, Barthelemy M, Vespignani A (2006) The role of the airline transportation network in the prediction and predictability of global epidemics. Proc Natl Acad Sci U S A 103: 2015-2020.
7. Lloyd AL, May RM (2001) Epidemiology. How viruses spread among computers and people. Science 292: 1316-1317.
8. McNamara JM, Barta Z, Fromhage L, Houston AI (2008) The coevolution of choosiness and cooperation. Nature 451: 189-192.
9. McNamara JM, Barta Z, Houston AI (2004) Variation in behaviour promotes cooperation in the Prisoner's Dilemma game. Nature 428: 745-748.
